# Acute Rheumatism

**Published:** 1871-05

**Authors:** W. M. Wells

**Affiliations:** Atlanta, Ga.


*Acute Rheumatism.* By W. M. Wells, M. D., Atlanta, Ga.

---

Perhaps no order of diseased action has received as many varied forms of treatment, as that of Acute Rheumatism.

The severity of the symptoms, both as regards pain and constitutional disturbance, which ordinarily continue several weeks, and the great tendency to develop organic heart disease, are quite sufficient to have produced that amount of solicitude and untiring efforts on the part of the profession, to discover some plan of treatment by which the duration of attack may be shortened, the pain alleviated, and the heart more surely guarded againt metastatic attack.

In the meantime, many able men have well-nigh abandoned the hope of such achievement, which has, in effect, to some extent, discouraged others of less acknowledged ability.

Dunglinson says, "the disease will generally run its course in spite of treatment; and, further, the disease rarely terminates in less than six weeks."

Warren would prescribe, "six weeks and two blankets."

Bennett says, "our treatment is purely empirical, and, as yet, no plan of treatment will do to rely upon."

By such statements, from such men, the young practitioner is too apt to be deterred from such investigation as may ultimately lead the profession to a more rational plan of treatment, the varying of which to meet emergencies being an easy task for the discriminating practitioner.

Inasmuch as the true pathology of the disease has not been settled, nor can be fully demonstrated, it follows that our opinions in regard to the same must be more or less speculative. Yet, there are *conditions* quite early developed, which are constant, which have much to do in perpetuating, if not in giving origin to the disease, and which conditions, I might further add, seem to make up the disease.

The abnormal condition, first to mention, (being, perhaps, of greatest importance) is "the lithic acid diathesis," in which condition that normal balance between the production of lithic acid in the system, and its excretion therefrom, is broken up, and excess from accumulation results.

There is, also, existing, or quite early developed, a strong tendency to local inflammation of the *fibrous* and fibroserous membranes.

Still another condition exists—that of hyperæsthesia of the sensory nerves. This last mentioned condition, I believe, as often results from local engorgement of some portion of the spinal column, or important ganglia, as from excess of lithic acid in the circulation. The latter, however, may serve to intensify and keep up pain, and, perhaps, both conditions react upon each other in the relation of cause and effect. Thus pain, by developing fever, may enhance the formation of lithic acid, which product in *excess* may in turn react unfavorably upon the nerve centres, as "a poison," thereby intensifying pain.

Lithic acid in excess, perhaps, plays another important part, that of *fixing* local inflammation upon the fibrous and fibroserous membranes, by the formation of such urates as are sparingly soluble upon the surface of such membranes, those being most subject whose secretions contain largely of albumen, soda and its chlorides, while mucous membranes seem to enjoy comparative immunity.

I think it highly probable that the quality of the *normal secretions* of synovial membranes, favors the local formation of urates of a semi-solid consistency ; which, from mechanical irritation, give rise to much pain and local inflammation.

This, I think, is well-nigh demonstrated in attacks of the larger joints, where, from the irritation by such deposits, the secretions are largely *augmented*, the joint thereby becomes swollen, by which distension the articulating surfaces are partially separated, and the pain is much alleviated ; and, in a short time, this excess of secretion re-dissolves the urates which are held in such solution as to be re-absorbed into the circulation, to be deposited in some other locality not yet invaded. Hence, the *metastatic* tendency, and, hence, the *duration* of attack.

The condition of hyperæsthesia always exists ; and pain seems to make up most of the trouble ; yet, it might be well to regard it more as a symptom, giving undeniable evidence of an abnormal condition of the blood, and often the existence of some local engorgement, by which nerve matter is *impinged* upon, and not necessarily engorgement, or inflammation of even modified character, existing in the *nerve material.*

Engorgements, with subsequent inflammation, always exists in and about the joints, too obvious to be overlooked ; yet, in addition, I have found in the cases coming under my care, that in almost every case, there was decided spinal tenderness from the outset, or was quite soon apparent, which condition we might reasonably look for, from the nature of exciting causes ordinarily ushering in attacks. Such, for instance, as exposures to vicissitudes of weather, exhaustive exercise, and such other agencies as rapidly overpower and exhaust nerve force ; spinal engorgements and congestions, supervening as consequent.

I would call the attention of the profession to this local trouble, as I believe it is too often overlooked ; and I further believe that early attention to such condition when it does exist, will very much shorten the duration of attack, modify the intensity, and largely aid in delaying cardiac complications.

But, to avoid being tedious, I will proceed to give the outlines of a plan of treatment which I have in many cases adopted, the results of which have been quite satisfactory. None of the cases have been protracted beyond ten days, while the average would be about six. One case yielded in the short space of three days. The treatment is best adapted to sthenic types of acute cases, and when the opposite obtains, the plan should be varied accordingly.

The indications I regard as being, first, the relieving of any local engorgement, if it exist. Secondly, the neutralizing and rapid elimination of *lithic acid.* Thirdly, the decided controlment of the *heart's action*; and the controlment of pain by such anæsthetic agents as are best calculated to do it, until the abnormal conditions giving rise to the same have been disposed of. The treatment in most cases should be premised by a mercurial purgative, which may to advantage be repeated on the second or third day, which, however, should in no case be pushed to a degree depletive, as it is of prime importance to husband the strength of the patient. If spinal tenderness exist, use cups freely, or blister over seat of trouble.

To fulfill the second (and very important indication) I have until latterly used the Salts of Potash—preferring the Nitrate—giving it in from ten to twenty grain doses, every two hours, taken in ice water. I am of late in the habit of using the Salts of Lithia, the Bromide being preferred, which I give in from three to five grain doses, every two hours, in solution, containing simple syrup. The facility with which the Salts of Lithia hold lithic acid in solution, should highly commend them to our use, and on which account I think they are destined to supplant the Salts of Potash in the treatment of Rheumatism, acute or chronic, and Gout, in all its forms.

To fulfill the third indication, I have found nothing equal to Tr. of Aconite and Hydrocyanic Acid blended. The Aconite being decidedly sedative to the heart's action, while the Prussic Acid is mainly sedative to the nerve centres, (and thereby a powerful anæsthetic) the specific action of both may be had at once. One an arterial, the other a nervous sedative, the mutual action of both almost prevents pain and fever, (and thereby prevents, to a very great degree, cardiac complications,) until sufficient time can be had to successfully depurate any excess of lithic acid, the prime cause in perpetuating the attack. I have rarely found it necessary to use any other anodyne than the sedative mixture just mentioned. I have in one case used fifteen grains Hydrate Chloral to the dose, given with the Aconite. The result was entirely satisfactory. In that combination the acid was, of course, left out.

As local applications to painful parts, I have used a liniment containing some Chloroform, Ext. Aconite, Tr. Arnica, and sufficient Oil to give consistency. I have, also, used, perhaps to better advantage, Kerosene Oil well applied, three times a day. But for its bad odor, I am better pleased with it than any other form of local applications.

After the grave symptoms have passed off, and there is but little fever, and but an occasional pain, I abandon the sedative mixture, and also lessen the amount of Potash, or Lithia, and give three times a day a pill containing Valeriante Quinia, Valeriante Iron, Macrotin, and Ext. Hyoscyami, adding a trace of the Extract Aconite, if there be any too great irritability of the heart. During the whole time, I order nourishing diet in sufficient quantity, and a brandy toddy at such times as there is absence of fever sufficient to warrant its use. I also allow a liberal quantity of Ice Water from the outset.

As a summary, I will briefly recapitulate as to the treatment, which consists mainly of but two prescriptions, which I continue until pain and fever subside, alternating the preparations ; except from time to time having to diminish, or increase, the sedative. I here append a few formulæ, such as I have ordinarily used:

No. 1. ℞—Potassæ Nitratis.................... ʒiv.<br>

Aquæ Dist.<br>

" Menth.......................... aa. ℥ii.<br>

Misce et fiat sol.<br>

S. Dessert-spoonful every two hours, in ice water.

No. 2. ℞—Aquæ Dist.......................... ℥ii.<br>

Tr. Aconite, Rad.................... gtt. lxiv.<br>

Med. Hydrocyanic Acid............. gtt. xxxii.<br>

Misce.<br>

S. Teaspoonful every two hours, till pain and fever subside.

No. 3. ℞—Lithia Bromidii..................... ʒi.<br>

Syrupi Simplex...................... ℥i.<br>

Aquæ Dist.......................... ℥ii.<br>

Misce et fiat sol.<br>

S. Dessert-spoonful every two hours, in water.

The third Recipe I very much prefer to the first, and should, I think, in most cases, take its plAce. The Prussic Acid in the second might be very well replaced by Hyrate of Chloral in from ten to fifteen grains to the dose.

